# Necroptosis-Related Genes Associated With Immune Activity and Prognosis of Colorectal Cancer

**DOI:** 10.3389/fgene.2022.909245

**Published:** 2022-06-16

**Authors:** Xinyi Peng, Zhili Xu, Yong Guo, Ying Zhu

**Affiliations:** ^1^ The First Clinical College of Zhejiang Chinese Medical University, Hangzhou, China; ^2^ Zhejiang Provincial Hospital of Traditional Chinese Medicine, Hangzhou, China

**Keywords:** colorectal cancer, necroptosis, immune microenvironment, prognostic model, drug sensitivity

## Abstract

This study aims at screening out the key necroptosis-related genes in colorectal cancer and elucidating the role of necroptosis-related genes in the immune activity and prognosis of colorectal cancer (CRC). The CRC patients’ data were downloaded from The Cancer Genome Atlas (TCGA). The non-negative matrix factorization method was applied to identify new molecular subgroups**.** Survival analysis and single sample Gene Set Enrichment Analysis were performed to determinate the differences in the overall survival time and immune status of the subgroups. Prognostic model was constructed on the basis of univariate Cox regression and LASSO analysis. Functional analyses were used to explore the potential mechanisms. Based on prognostic related necroptosis genes, we identify two molecular subgroups with significantly different survival. The better prognosis was associated with more active immune infiltration and upregulated expression of immune checkpoints. We screened nine necroptosis related genes as key prognostic genes and established a risk model, which showed a good potential for survival prediction in colorectal cancer. Nomogram assessment showed that the model had high reliability for predicting the prognosis of colorectal cancer patients. The high-risk and low-risk group also has different sensitivity to immunotherapy and commonly used drugs for colorectal cancer. Overall, necroptosis related genes were involved in the immune microenvironment of colorectal cancer patient, could be utilized to predict the prognosis of colorectal cancer and develop more individualized treatment.

## Introduction

Colorectal cancer (CRC) is the second most common malignant tumor and accounts for the fourth cancer-related death worldwide (Bray et al., 2018). At present, the main treatment methods of CRC are surgery, radiotherapy, chemotherapy, targeted therapy, and immunotherapy (Dekker et al., 2019). Although the current treatment solutions have extended the survival time of patients with CRC, the prognosis of patients is still not ideal, for example, 3-years survival for stage IV colorectal cancer in England was only 23.5% (Benitez Majano et al., 2019). One of the main reasons is the drug resistance of cancer cells, and about 80%–90% of deaths in cancer patients directly or indirectly resulted from drug resistance (Yuan et al., 2017). At present, there is still a lack of effective strategies to eliminate drug resistance in tumor cells.

Necrosis and apoptosis are two classic and famous ways of cell death. It is by promoting apoptosis that the majority chemotherapy works (Su et al., 2016). However, apoptosis dysfunction is regarded as a crucial intrinsic reason of chemoresistance, leading to tumor progression and chemotherapy failure (Hanahan and Weinberg, 2011; Holohan et al., 2013). Necroptosis was discovered as a novel form of programmed cell death, mainly mediated by Receptor-Interacting Protein 1 (RIP1), RIP3, and Mixed Lineage Kinase Domain-Like protein (MLKL) (Degterev et al., 2005; Linkermann and Green, 2014). Its mechanism is similar to apoptosis and morphology is similar to necrosis (Christofferson and Yuan, 2010). These finding suggested that necroptosis might provide a promising approach of inducing cancer cell death avoiding apoptosis resistance and play a vital role in the regulation of cancer biology (Gong et al., 2019). Koo et al. (2015) reported RIPK3 was decreased or even absent in some cancer cell lines. This downregulation was proven to be correlated with tumor progression and a worse prognosis (Feng et al., 2015; Koo et al., 2015; Bozec et al., 2016). Zhao et al. (2021) summarized necroptosis-related lncRNAs and established a novel model for prognostic prediction in gastric cancer.

Some studies also have demonstrated that necroptosis is involved in the tumorigenesis and metastasis of CRC. Wang et al. (2019) proved that necroptosis regulated the tumor repopulation in colorectal cancer after radiotherapy by activating the RIP1/RIP3/MLKL/JNK/IL8 pathway. However, there are currently no models based on the protein-coding RNA of necroptosis. Here, we constructed an efficient and prognostically significant model consisting of necroptosis signatures, established a promising nomogram to predict the prognosis of CRC, and assess the relationship between necroptosis and drug sensitivity in CRC.

## Methods

### Data Acquisition

**FIGURE 1:**
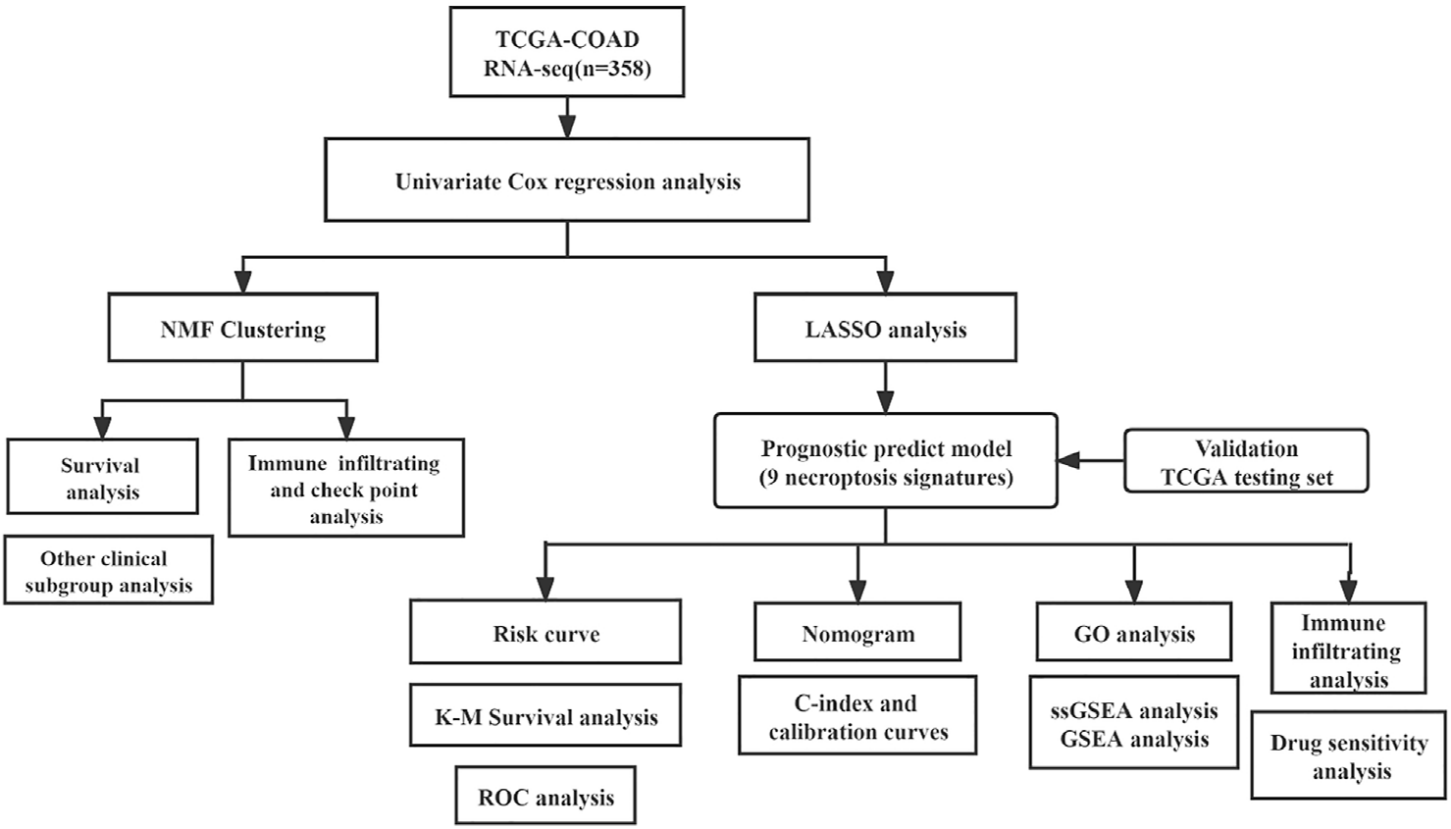
Flowchart for profiling the necroptosis-related genes of colorectal cancer.

The RNA-Seq data and corresponding clinical information were downloaded from The Cancer Genome Atlas (TCGA; https://tcga-data.nci.nih.gov/tcga/). Only cases with the primary colon tumor and overall survival (OS) more than 30 days were included in our study. The expression levels of fragments per kilobase of exon model per million reads mapped (FPKM) data were log-transformed by log2 (FPKM +1) for subsequent analysis. The necroptosis-related genes (NRGs) were obtained from the KEGG database (Kyoto Encyclopedia of Genes and Genomes, https://www.kegg.jp/) and previous references (Zhao et al., 2021) (Supplementary Table S1). And the flow chart of the analysis work is shown in Figure 1.

### Identification of Prognosis-Related NRGs and Non-Negative Matrix Factorization

Prognosis-related NRGs are preliminarily identified by univariate Cox regression analysis, using the “survival” package. Then based on these prognosis-related NRGs, all COAD samples would be divided into subgroups *via* Non-Negative Matrix Factorization (NMF) method, which can effectively reduce the dimension of RNA-seq data and help to find subgroups with similar gene expression patterns intra groups.

### Immune Analysis for Subgroups

With “survminer” and “survival” packages, Kaplan–Meier (K-M) survival curve analysis was performed to estimate the difference in overall survival (OS) time of subgroups. The differences in clinical characteristics (age, gender, T, N, M, stage) and the expression of prognosis related NRGs were visualized by a heat map based on “pheatmap” package. We calculated the scores of immune microenvironment by ESTIMATE algorithm and researched the differences in the activity of immune infiltrating cells and 30 immune checkpoints of subgroups, using “estimate,” “GSVA” and “GSEABase” package.

### Construction and Validation of the Risk Model

The prognostic prediction ability of NRGs was further explored. First, we randomly divided all samples into training and validation groups at a 1:1 ratio by “caret” package. Then, on the basis of the result of univariate Cox, we used the least absolute shrinkage and selection operator (LASSO) analysis to exclude the over-fitting NRGs through the “glmnet” package. Thirdly, we used the formula $risk\;score=\sum_{k=1}^{n}\left(coef\;\left(Gene\;k\right)\times \exp\;\left(Gene\;k\right)\right)$ to calculating the risk score. Exp (Gene) means the expression level of the included NRGs, and coef (Gene) means corresponding coefficients. According to the risk score, all samples would be divided into high-risk and low-risk subgroups based on the median value.

Survival analysis, the time-dependent receiver operating characteristic (timeROC) and the area under the curve (AUC) were drawn by “survival,” “survminer” and “timeROC” packages.

### Independence Test of Predictive Ability and Construction of the Prognostic Nomogram

Univariate and multivariable Cox regression analyses were used to confirm whether the risk score could serve as an independent prognostic factor with the “survival” package, and the results were visualized in the form of forest maps. Based on the clinical features and gene signature, we established a nomogram and assessed the OS probability at 1, 3, and 5 years for patients. We also calculated the Concordance index (C-index) of this predictive model and drew calibration curves. These results could reflect the relationship between the observed values and the optimized values and evaluate the prediction ability of this model. The “rms” package was applied in this process.

### Functional Enrichment Analysis and Immune-Related Analysis

First, we conducted Gene Ontology (GO) enrichment analysis for genes in the risk model. Then, single sample Gene Set Enrichment Analysis (ssGSEA) and Gene Set Enrichment Analysis (GSEA) were performed to evaluate the activity of Kyoto Encyclopedia of Genes and Genomes (KEGG) and Hallmarks pathways within the high-risk and low-risk groups, *p*-value <0.05 and FDR <0.05 were considered statistically significant. The pathways of KEGG and Hallmarks gene sets were download from the MsigDB website (http://www.gsea-msigdb.org/gsea/msigdb/collections.jsp). We also investigated the different immune infiltration status between two groups based on ssGSEA algorithm. “limma,” “clusterProfiler” and “GSVA” packages were used in this process.

In order to assess the immunotherapy response capacity of samples, we used the tumor immune dysfunction and exclusion (TIDE) algorithm one the TIDE website (http://tide.dfci.harvard.edu) to calculate the TIDE score of each group.

### Drug Sensitivity Prediction

Drug sensitivity data of 60 cell lines were obtained from the CellMiner database (https://discover.nci.nih.gov/cellminer/loadDownload.do), and drugs commonly used in colorectal cancer were screened for drug sensitivity analysis. We used “oncoPredict” packages to predict the IC50 of these drugs, further mining the clinical significance of the risk model (Maeser et al., 2021).

### Statistical Analysis

The quantitative data were expressed as mean ± SD, and the qualitative data were expressed as number (ratio%). For the quantitative data, the comparison between the two groups was performed by *t*-test (normal distribution data) or Wilcoxon-test (non-normal distribution data). For the qualitative data, the comparison between the two groups was performed by Chi-square analysis and Fisher’s test. R software (version 4.1.2) was applied for statistical analyses.

## Results

### Identification of Prognostic Necroptosis-Related Genes

**FIGURE 2:**
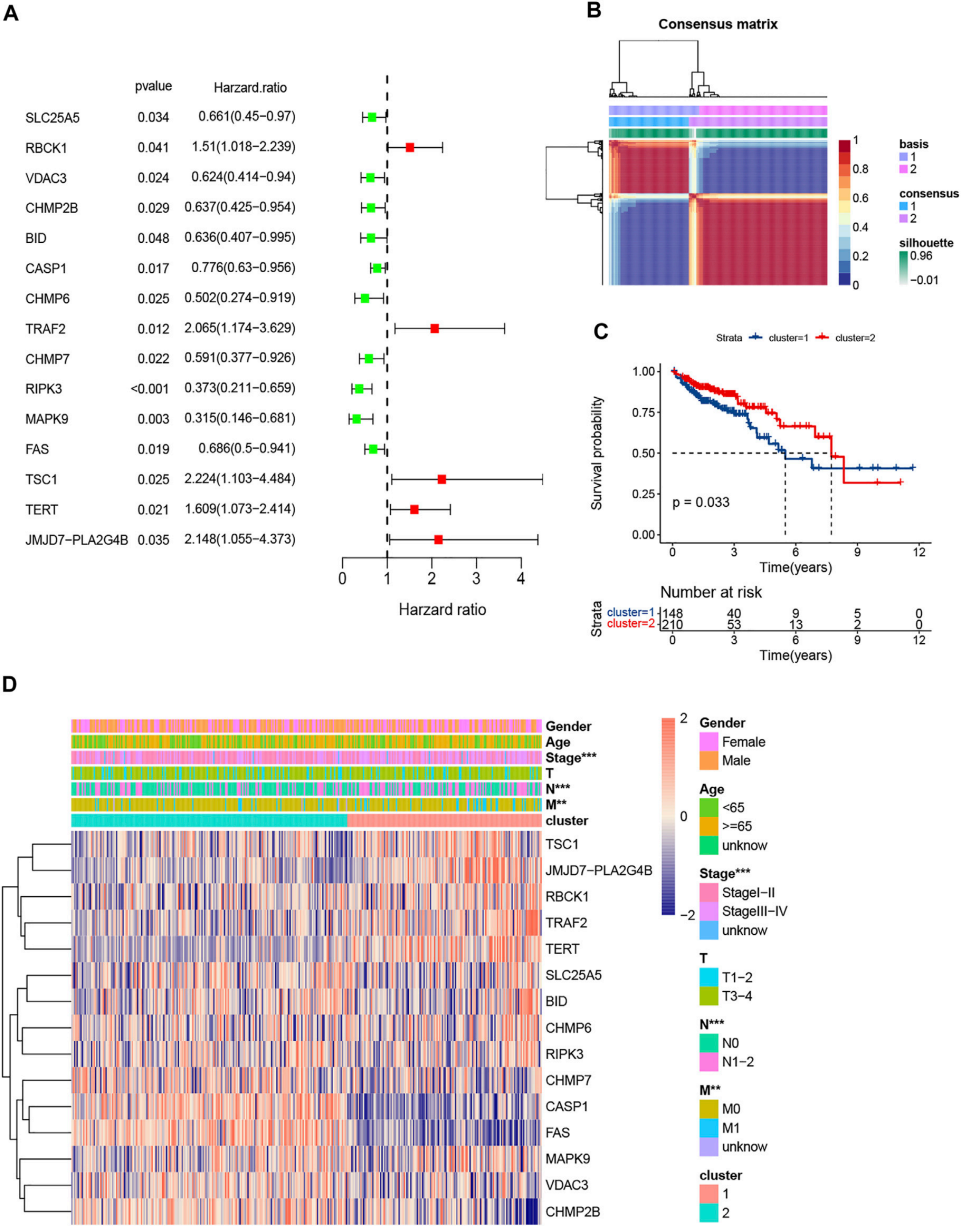
Screening of prognosis-related NRGs by univariate Cox and clustering by nonnegative matrix factorization (NMF). **(A).** The forest plot of 15 prognosis-related NRGs (*p* < 0.05); **(B).** Colorectal cancer samples were clustered by NMF method; **(C).** Survival analysis of two clusters; **(D).** Heatmap and clinicopathologic features of Clusters 1 and 2.

A total of 358 patients with primary colorectal cancer who were followed for more than 1 month were included in the study. Then, we obtained 204 NRGs from KEGG and previous references (supplement 1). In total, 15 prognosis-related NRGs were screened by Univariate Cox regression (Figure 2A).

### Non-Negative Matrix Factorization Cluster and Immune Analysis for Subgroups

The NMF clustering approach was applied to separate all CRC patients into 2 subgroups (Figure 2B). 148 patients were clustered into Cluster 1 and the other 210 patients into Cluster 2. Survival analysis indicated Cluster 2 had better overall survival than Cluster 1 (*p* < 0.001; Figure 2C). The expression levels of the NRGs, lymph node (N) stage, metastasis (M) stage and pathologic stage in the two subgroups were different (Figure 2D).

**FIGURE 3:**
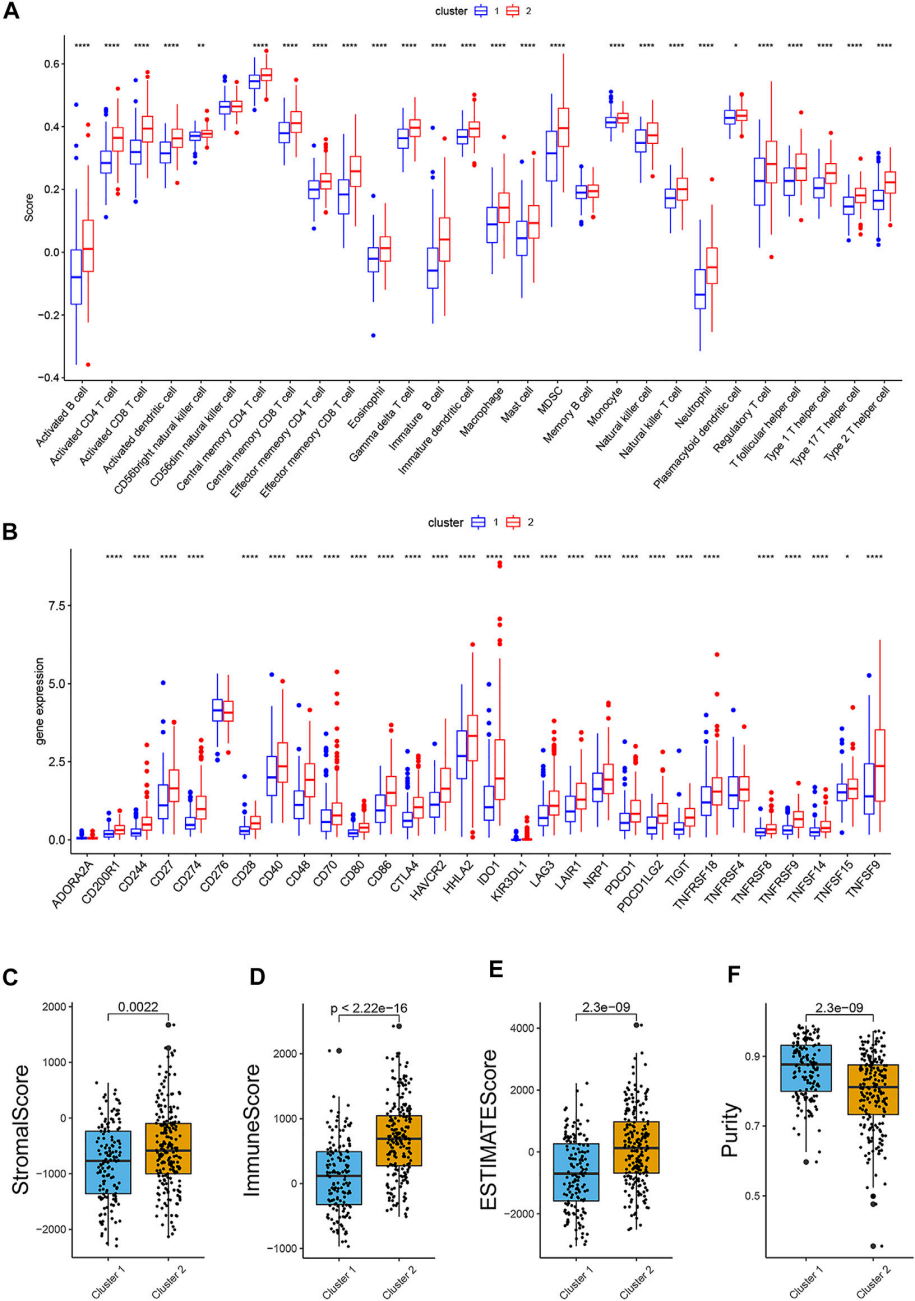
Comparison of immune infiltration, expression level of immune checkpoint and ESTIMATE scores in two groups. **(A).** Boxplot of the difference of immune infiltration cells in two clusters; **(B).** Boxplot of the expression level of immune checkpoint in two clusters; **(C).** Stromal score; **(D).** Immune score; **(E)**. ESTIMATE score; **(F)**. Purity. (*p* < 0.05 *, *p* < 0.01 **, *p* < 0.001 ***, *p* < 0.0001 ****).

Moreover, the outcome of immune infiltration analysis showed that, a total of 26 immune cells were significantly more active in Cluster 2, except for CD56dim natural killer cell and memory B cell (Figure 3A). Simultaneously, the expression level of 27 immune checkpoints in Cluster 2 were also higher than that in Cluster1, especially PD-L1 and CTLA-4 (Figure 3B).

### Construction and Validation of the Risk Model

**FIGURE 4:**
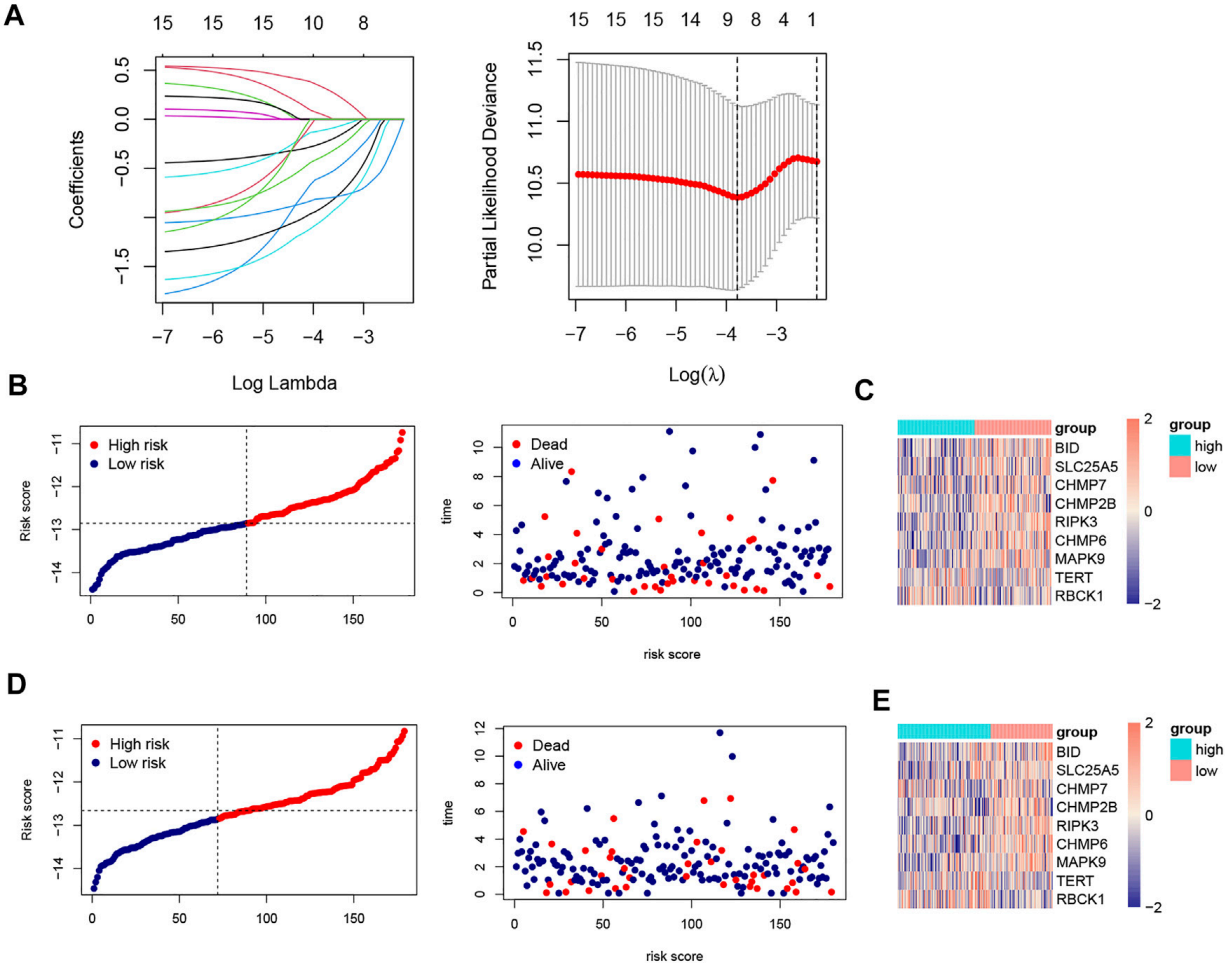
LASSO analysis, risk score plots and expression of 9 NRGs in training and validation sets. **(A).** Construction of prognostic model based on LASSO Cox analysis; **(B)**. The distribution of patients’ survival times and survival status in training set; **(C)**. Heatmap of the expression matrix of 9 genes in training set; **(D).** The distribution of patients’ survival times and survival status in validation set; **(E)**. Heatmap of the expression matrix of 9 genes in validation set.

All samples were randomly divided into training and validation sets at a 1:1 ratio, the clinical characteristics of two sets were shown in Table 1. Then, we conducted the LASSO analysis based on univariate Cox results, and finally identified 9 genes and corresponding coefficients to construct the risk model (Table 2). The calculation formula of risk score was as follows, risk score = (−0.235326902*SLC25A5 exp.) + (0.318710455 * RBCK1 exp.) + (−0.574946213 * CHMP2B exp.) + (−0.108698734 * BID exp.) + (−0.885564522 * CHMP6 exp.) + (−0.370269376 * CHMP7 exp.) + (−0.805281486 * RIPK3 exp.) + (−1.012280584 * MAPK9 exp.) + (0.04072479 * TERT exp.). All samples were divided into high-risk and low-risk subgroups based on the median value (Figure 4B). The heatmap showed that most of these genes were downregulated in the high-risk group (SLC25A5, CHMP2B, BID, CHMP6, CHMP7, RIPK3, and MAPK9), whereas the others were upregulated (TERT and RBCK1) (Figures 4C,E). As shown in heatmaps, most of these genes were downregulated in the high-risk group, suggesting that inhibition of the necroptosis-related pathways may imply a better prognosis in CRC. Overall survival time revealed that the high-risk group obviously had a worse prognosis (*p* < 0.0001). In the same way, these conclusions could be drawn in the validation set and overall samples as well.

**TABLE 1:** Characteristics of patients in the training and validation set.

	Total (*n* = 358)	Training set (*n* = 178)	Validation set (*n* = 180)	*p*-value
Age				
<65	145 (40.5%%)	72 (40.4%)	73 (40.6%)	1.000
≥65	212 (59.2%)	106 (59.6%)	106 (58.9%)	
Unknown	1 (0.3%)	0 (0.0%)	1 (0.6%)	
Gender				
Female	161 (45.0%)	79 (44.4%)	82 (45.6%)	0.453
Male	197 (55.0%)	99 (55.6%)	98 (54.4%)	
T stage				
T1	10 (2.8%)	6 (3.4%)	4 (2.2%)	0.184
T2	66 (18.4%)	29 (16.3%)	37 (20.6%)	
T3	245 (68.4%)	119 (66.9%)	126 (70.0%)	
T4	37 (10.3%)	24 (13.5%)	13 (7.2%)	
N stage				
N0	211 (58.9%)	107 (60.1%)	104 (57.8%)	0.311
N1	84 (23.5%)	45 (25.3%)	39 (21.7%)	
N2	63 (17.6%)	26 (14.6%)	37 (20.6%)	
M stage				
M0	301 (84.1%)	151 (84.8%)	150 (83.3%)	0.499
M1	52 (14.5%)	26 (14.6%)	26 (14.4%)	
Unknown	5 (1.4%)	1 (0.6%)	4 (2.2%)	
Pathologic stage				
Stage Ⅰ	62 (17.3%)	28 (15.7%)	34 (18.9%)	0.735
Stage Ⅱ	135 (37.7%)	72 (40.4%)	63 (35.0%)	
stage Ⅲ	98 (27.4%)	48 (27.0%)	50 (27.8%)	
stage Ⅵ	52 (14.5%)	26 (14.6%)	26 (14.4%)	
Unknown	11 (3.1%)	4 (2.2%)	7 (3.9%)	

**TABLE 2:** Prognostic genes generated by LASSO Cox analysis.

Gene	Full Name	Coef
SLC25A5	Solute carrier family 25 member 5	-0.235326902
RBCK1	RANBP2-type and C3HC4-type zinc finger containing 1	0.318710455
CHMP2B	Charged multivesicular body protein 2B	-0.574946213
BID	BH3 interacting domain death agonist	-0.108698734
CHMP6	Charged multivesicular body protein 6	-0.885564522
CHMP7	Charged multivesicular body protein 7	-0.370269376
RIPK3	Receptor interacting serine/threonine kinase 3	-0.805281486
MAPK9	Mitogen-activated protein kinase 9	-1.012280584
TERT	Telomerase reverse transcriptase	0.04072479

**FIGURE 5:**
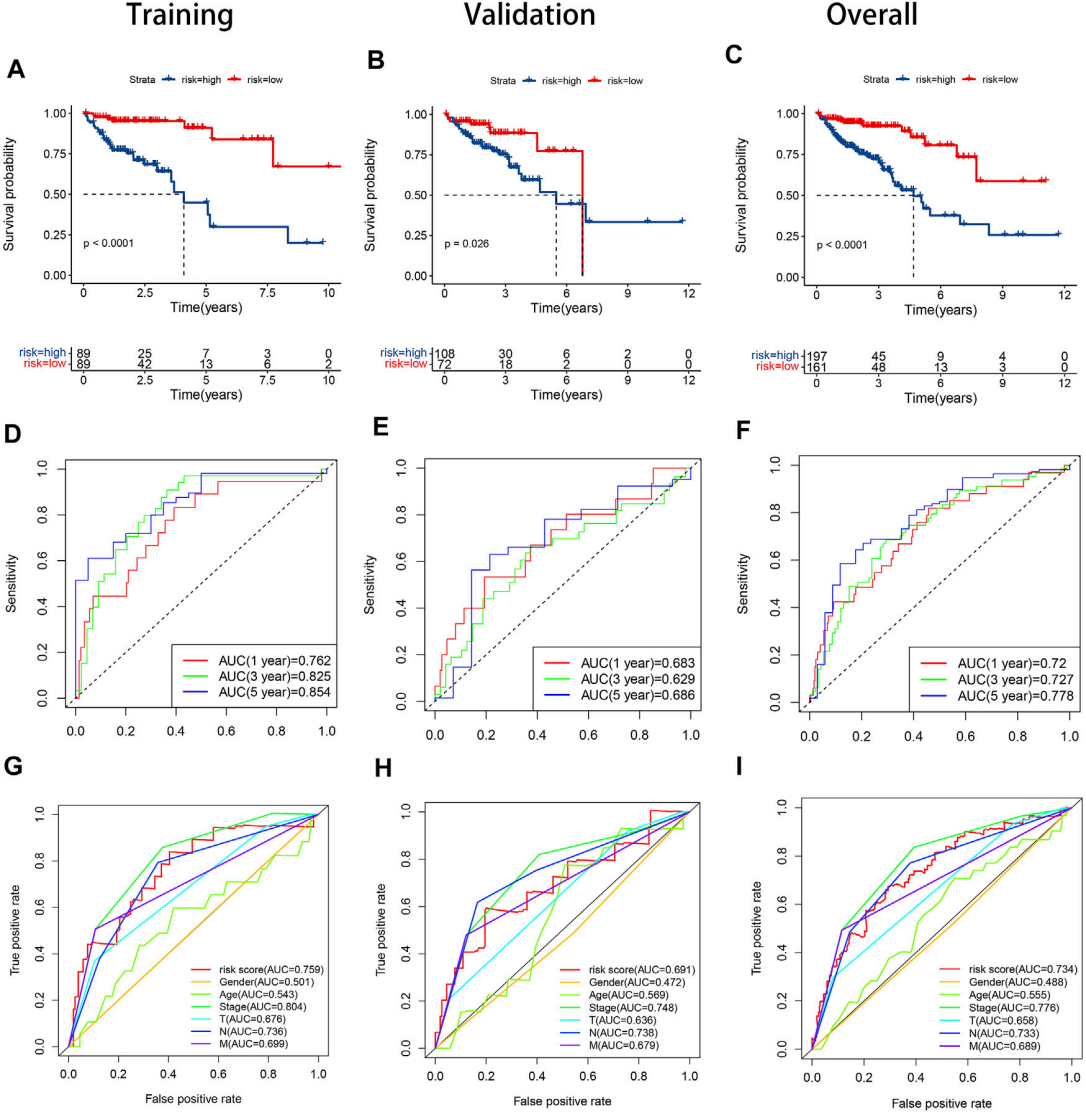
KM curves, time-dependent ROC curves and clinical feature ROC curves in the training set, validation set and overall samples respectively. **(A).** KM curve in the training set; **(B).** KM curve in the validation set; **(C).** KM curve in the overall samples; **(D).** Time-dependent ROC curve in the validation set; **(E).** Time-dependent ROC curve in the validation set; **(F).** Time-dependent ROC curve in the validation set; **(G).** Clinical feature ROC curve in the overall samples; **(H).** Clinical feature ROC curve in the overall samples; **(I).** Clinical feature ROC curve in the overall samples.

In the training set, the AUCs were 0.762, 0.825, and 0.854 at 1, 3, and 5 years respectively. In the validation set, the AUCs were 0.683,0.629, and 0.686 at 1, 3, and 5 years respectively. And in all samples, the AUCs were 0.720, 0.727, and 0.778 at 1, 3, and 5 years respectively (Figures 5D–F). The ROC curve and AUC value of clinical factors were shown in Figures 5G−I.

### Independent Prognostic Analysis and Construction of the Prognostic Nomogram

**FIGURE 6:**
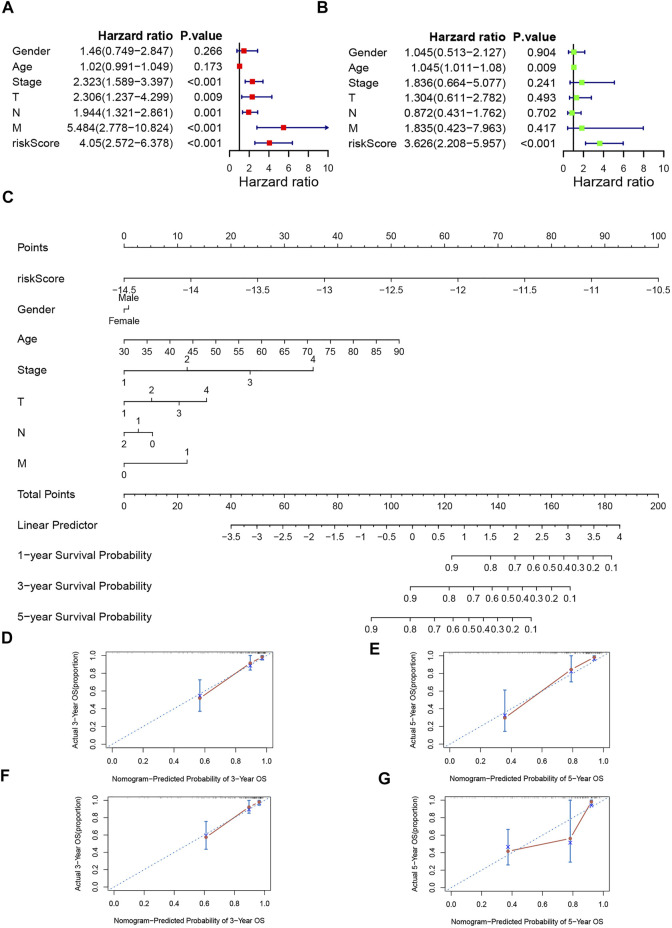
Independence veridiction of the risk model and construction and calibration of nomogram. **(A).** Univariate independent prognostic analysis in the training group; **(B).** Multivariate independent prognostic analysis in the training group; **(C).** A nomogram integrating clinical factors and risk score; **(D,E).** Calibrations of the nomogram at 3-years and 5-years survival in the training set; **(F,G).** Calibrations of the nomogram at 3-years and 5-years survival in the validation set.

We utilized univariate and multivariable Cox regression to confirm whether the risk score could serve as an independent prognostic factor. The results of univariate Cox regression displayed that stage, T stage, N stage, M stage, and risk score were independent predictive factors (Figure 6A). And according to the results of multivariable Cox regression, age and risk score were independent predictive factors (Figure 6B). We also created a nomogram and assessed the 3 and 5- years OS probability for patients (Figure 6C). The C-index of training set and validation set were 0.831 and 0.794 respectively, and the 3- and 5-years OS predicted value for both groups were close to the corresponding actual OS values, which indicates a remarkable ability for survival prediction with the nomogram. (Figures 6D,E).

### Immune Infiltration Analysis and Immunotherapy Response Analysis

**FIGURE 7:**
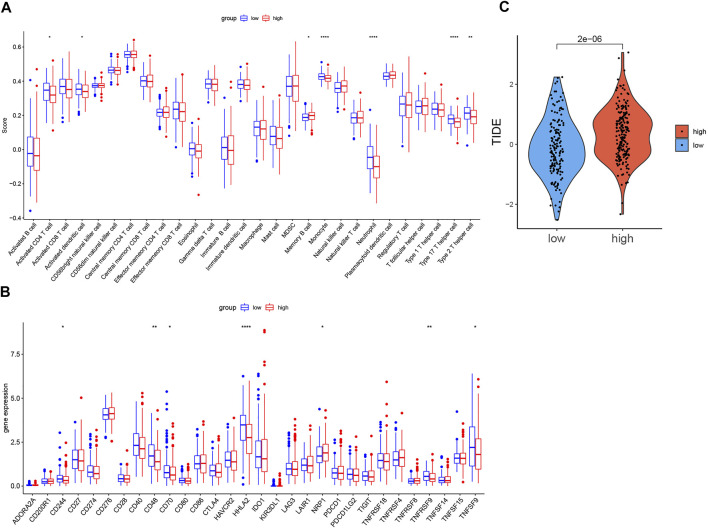
Comparison of immune infiltration, expression level of immune checkpoints, TIDE score in two groups. **(A).** Boxplot of the difference of immune infiltration cells in high- and low-risk groups; **(B).** Boxplot of the expression level of immune checkpoint in high- and low-risk groups. **(C).** Boxplot of the TIDE score in high- and low-risk groups (*p* < 0.05 *, *p* < 0.01 **, *p* < 0.001 ***, *p* < 0.0001 ****).

The comparison of the activity of each immune cell and the expression of immune checkpoints in the high-risk and low-risk groups was shown in box plots (Figures 7A,B). In the high-risk group, the activity of the activated CD4^+^ cell, activated dendritic cell, monocyte, neutrophil, Type 17 T helper cell, and Type 2 T helper cell significantly decreased, while the activity of memory B cell increased. The expression of D244, CD48, CD70, HHLA2, TNFRSF9, and TNFSF9 was lower in the high-risk group, but the expression of NRP1 was higher. Although there was no statistical difference in PD-L1 and CTLA-4, the high-risk group showed a downward tendency.

As Figure 7C shows, the TIDE score of the low-risk group was significantly lower than that of the high-risk group.

### Functional Enrichment Analyses

**FIGURE 8:**
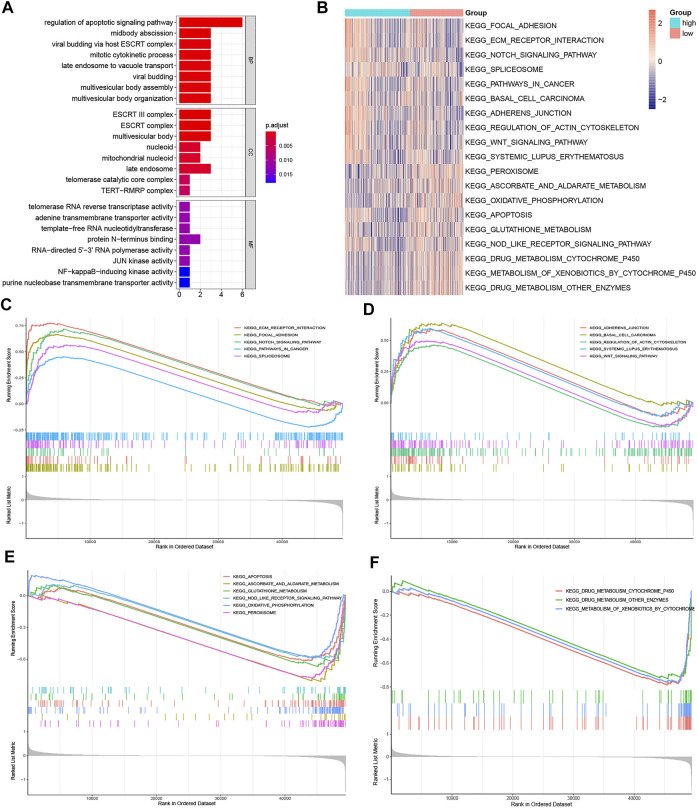
Gene function enrichment analyses. **(A).** The signaling pathways enriched by necroptosis genes signatures through GO analysis; **(B).** The heatmap of ssGSEA of the KEGG gene sets; **(C,D).** KEGG enrichment analysis in the high-risk group; **(E, F).** KEGG enrichment analysis in the low-risk group.

**FIGURE 9:**
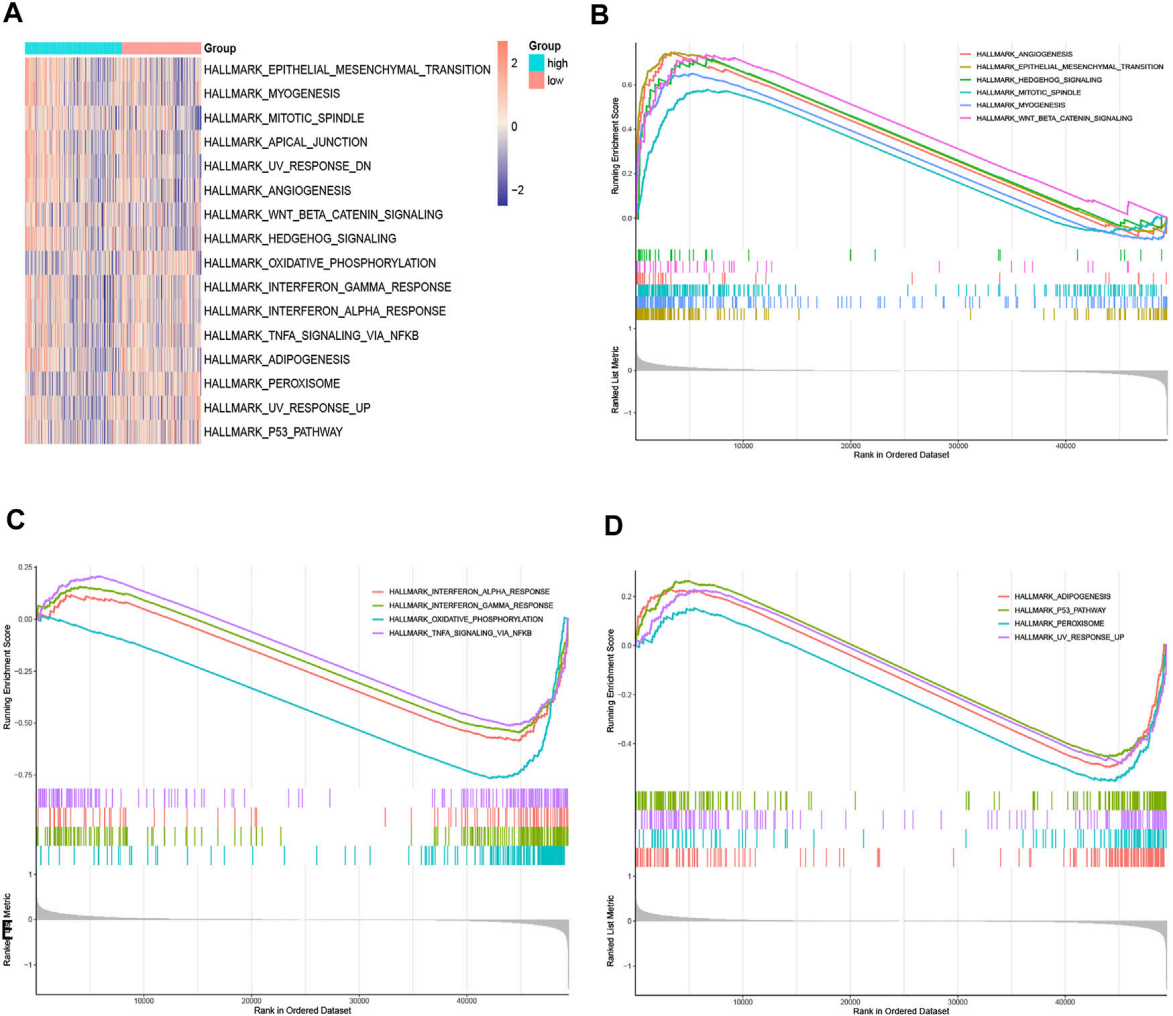
Gene function enrichment analyses. **(A).** The heatmap of ssGSEA of the hallmark gene sets; **(B,C).** Hallmark enrichment analysis in the high-risk group; **(D).** Hallmark enrichment analysis in the low-risk group.

The possible underlying mechanisms that NRGs affected the prognosis of CRC patients were further detected using functional enrichment analyses. GO analysis reflected that NRGs were closely associated with processes of cell cycle regulation and cell proliferation, such as in the regulation of apoptotic signaling pathway, midbody abscission, ESCRT III complex, and telomerase RNA reverse transcriptase activity (Figure 8A). The comparison of KEGG and cell hallmarks pathway activity in high-risk and low-risk groups was visualized in heatmaps basing on ssGSEA analysis (Figures 8B, 9A). And further GSEA analyses revealed two groups are different in some tumor-, drug metabolism- and energy metabolism-related pathways (Figures 8C–E, 9B–D). Pathways in cancer, ECM receptor interaction, epithelial mesenchymal transition and angiogenesis were significantly enhanced in the high-risk group. And oxidative phosphorylation, P53 pathway and Interferon-gamma (INF-γ) and alpha (INF-α) response were significantly activated in the low-risk group.

### Drug Sensitivity Prediction

**FIGURE 10:**
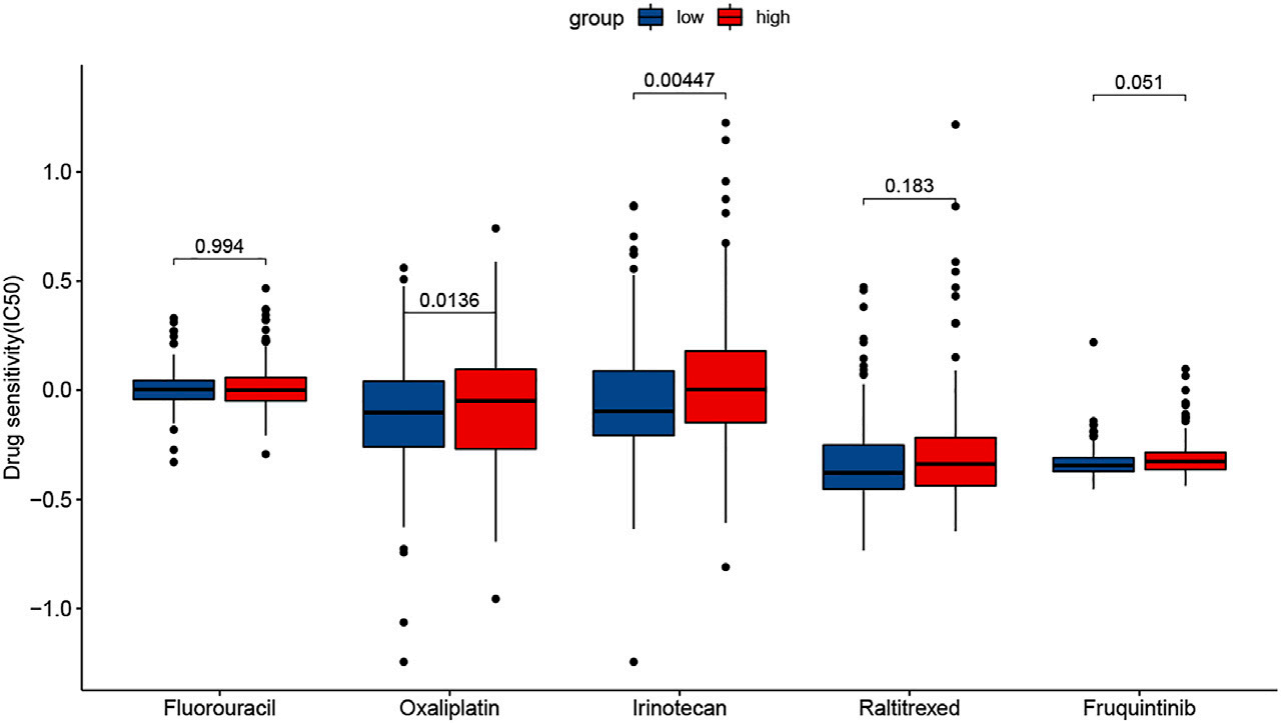
Prediction of drug sensitivity in the therapy of colorectal cancer.

The results showed that the sensitivity to two drugs differed significantly. The low-risk group had a higher sensitivity to Oxaliplatin and Irinotecan (Figure 10). In addition, the sensitivity to Fluorouracil was very similar in two groups.

## Discussion

Colorectal cancer (CRC) is one of the top5 most common malignant tumors in the world. Although individualized treatment for CRC keeps improving, morbidity and mortality of CRC still remain high (Dekker et al., 2019). Recently, many studies have explored the role of cell death modes in oncogenesis and development of CRC, including necroptosis, autophagy and ferroptosis, expecting to find a novel anti-cancer approach for CRC (Gong et al., 2019; Lei et al., 2021; Wang et al., 2021). Necroptosis, as a non-Caspase-dependent programmed cell death with necrosis characteristics, has attracted extensive attention in the study of multiple cancers (Gong et al., 2019; Ando et al., 2020). Several studies elucidated that necroptosis plays an important role in colorectal cancer. Feng et al. (2015) found that overexpression of RIP3 significantly suppressed the proliferation of CRC cells. Li et al. (2017) reported that low expression of MLKL was correlated with poor overall survival in CRC. However, the role of necroptosis in CRC is not entirely clear and remains to be studied. Therefore, we try to systematically research the relationship between the NRGs, the prognosis and the immune microenvironment and drug sensitivity, providing new ideas for necroptosis-related prognostic prediction and targeted therapy in CRC.

In this study, we firstly clarified 15 prognostic related NRGs of CRC by univariate Cox regression analysis. Based on these genes, we identify two molecular subgroups. The Cluster 2 had better prognosis, more active immune infiltration and higher expression of immune checkpoints, including PD-L1and CTLA-4. These results revealed that necroptosis-related genes were strongly associated with the tumor microenvironment of CRC, which may serve as new biomarkers for screening the beneficiaries for immunotherapy. Then based on the 9 NRGs selected by LASSO analysis, we established a risk model, which showed a good potential for survival prediction in CRC. A nomogram integrating clinical characteristics and risk score also had high reliability for predicting the prognosis, in which the risk score has the highest weighted score, followed by age and stage. Additionally, the analysis indicated that the risk model could be an independent predicted factor.

The risk model consisted of 9 prognostic related NRGs: SLC25A5, RBCK1, CHMP2B, BID, CHMP6, CHMP7, RIPK3, MAPK9 and TERT. Among these 9 NRGs, most of them have been reported to be closely associated with the development and prognosis of CRC or other malignancies. The full name of RIPK3 is receptor-interacting protein kinase 3, one of the key mediators in necroptosis, also known as a key component of necrosomes (Liu et al., 2021). RIPK3 can interact with RIPK1 to form necrosome and phosphorylate mixed lineage kinase domain-like (MLKL) (Gong et al., 2019). In addition, a prior study has noted that its upregulation is related to better DFS and OS of colorectal cancer (Feng et al., 2015). SLC25A5 has been demonstrated to attenuate cell proliferation and promote programmed cell death-related genes expression, and be positively correlated with the OS of colorectal cancer (Chen et al., 2022). As for RBCK1, Liu et al. (2019) found the overexpression of it could promote the chemoresistance and lead to poor prognosis in colorectal cancer. TERT participated in the early stages of colorectal cancer development through inducing COX-2 expression to influence the tumor’s stromal microenvironment (Ayiomamitis et al., 2019). MAPK9 belongs to the MAPK pathway, it has been reported that genetic variation in the MAPK signaling pathway genes can mediate cell proliferation and migration of colorectal cancer cells (Schwartsmann et al., 2005). CHMP7 plays a significant role in the endosomal sorting pathway, and the deficient expression of CHMP7 in the majority of tumor tissues has been reported (Guo Y. et al., 2021). However, the role of CHMP2B, BID and CHMP6 in colorectal cancer have not been reported yet, which may provide new biomarkers for future research on the relationship between necrosis and colorectal cancer.

By performing enrichment analyses, the functions of these NRGs were explored. The GO analysis showed that these genes may influence cell cycle regulation and programmed death, such as mitotic cytokinetic process, purine nucleobase transmembrane transporter activity, ESCRT III complex and adenine transmembrane transporter activity. These pathways are consistent with the characteristic of necrotic cell death, which is the degradation of lysosomal membrane and vacuolation of cytoplasm would results in rupture of the cell membrane and cell lysis (Murphy et al., 2013; Cai et al., 2014). The GSEA analysis also showed that, ECM receptor interaction, epithelial mesenchymal transition and angiogenesis were significantly upregulated while oxidative phosphorylation, glutathione metabolism, TNF-αsignaling pathway and P53 pathway and were downregulated in the high-risk group, which were closely associated with tumorigenesis, tumor metabolism, and metastasis (Chasov et al., 2021).

Additionally, analysis of immune infiltration status indicated that activated CD4^+^ T cell, activated dendritic cell, monocyte, neutrophil, Type 17 T helper cell (Th17), and Type 2 T helper cell (Th2) infiltration level decreased but memory B cell increased in the high-risk group. B cells, as the main effector cells of the adaptive immune response, reported that increased B cell count is related to better clinical prognosis of CRC (Edin et al., 2019). And previous studies have proven that the immune cell infiltration features with a better prognosis were marked by high T cells in CRC patients (Guo J.-N. et al., 2022; Lundberg et al., 2021; Zinovkin et al., 2021). CD4^+^ T cells are indispensable in orchestrating antitumor immunity (Ben Khelil et al., 2022). Various subsets of CD4^+^ T cells play multifaceted roles in the cancer response, including Th2, and Th17 (Galon and Bruni, 2020). Th17 cells could promote anti-tumor immune response by recruiting immune cells into the tumor microenvironment, inducing effector CD8^+^ T cells, or transforming to the Th1 phenotype and producing abundant IFN-γ (Guéry and Hugues, 2015). The impairment of dendritic cell functions is decisive for immune evasion, tumor growth, metastasis initiation, and treatment resistance in CRC (Legitimo et al., 2014; Subtil et al., 2021). These results were consistent with our study. Accumulation evidence indicated that immunogenic or inflammatory characteristics driven by necroptosis can influence anti-tumor immunity (Sprooten et al., 2020; Scarpitta et al., 2021). Necroptotic cells secrete various chemokines to recruit efficient myeloid or granulocytes, for instance, CXCL8, CXCL1, and CXCL2 (Zhu et al., 2018). It is demonstrated that necrotic cells showed rapid and sustained attraction towards monocytes, dendritic cells, and neutrophils (Garg et al., 2017). Hoecke et al. (Van Hoecke et al., 2018) confirmed the direct intratumoral delivery of MLKL-mRNA coding could rapidly induce CD4^+^ T cells to prevent tumor progression, and this process depends on Type I interferon signaling and Batf3-dependent dendritic cells. These may be the underlying mechanisms for the different immune landscapes and good prognosis in the low-risk group.

The expression of D244, CD48, CD70, HHLA2, TNFRSF9, and TNFSF9 was lower in the high-risk group, but the expression of NRP1 was higher. Although there was no statistical difference in PD-L1 and CTLA-4, the high-risk group showed a downward tendency. The outcome suggested that the necroptosis risk model can be used as a predictive marker of the efficacy of some immune checkpoint inhibitors. And the result of the comparison of the TIDE score in two groups showed that the low-risk group is more sensitive to immunotherapy, which is consistent with the status of active immune cells and high expression of immune checkpoints in the low-risk group.

According to the analysis of drug sensitivity, the low-risk group had a higher sensitivity to Oxaliplatin and Irinotecan and the sensitivity to Fluorouracil was very similar in the two groups. We speculated that the low-risk group would benefit from using FOLFOX and FOLFIRI among many chemotherapies, while the high-risk group might be resistant to these drugs. And necroptosis may be involved in the development of drug resistance, which needs more research to explain the potential metabolisms.

To confirm the advantages of our model, we selected 2 prognostic risk models, the 6-long-non-coding RNAs (lncRNA) (Liu et al., 2022) and 7-microRNA (miRNA) signatures (Huang et al., 2021), which were both necroptosis-related gene models in colorectal cancer. The 1-, 3-, and 5-years AUCs of the 6-lncRNA signature were 0.719, 0.716, and 0.724, respectively. And the 1- and 5-years AUCs of 7-miRNA signature were 0.659 and 0.626, respectively. The AUCs of these models were all lower than those of our 9-gene model, indicating that the performance of our model was better.

Inevitably, there are some limitations in our study. First, as the data was incomplete, this model didn’t include some other clinical information in the assessment, for example, the state of RAS and BRAF. Second, all the conclusions of this study were based on bioinformatics analysis, lack of the support from experiment validation. Third, as the relationship between necroptosis and colorectal cancer largely remains unclear, the underlying mechanisms of necroptosis in colorectal cancer need further explorations.

## Data Availability

The original contributions presented in the study are included in the article/Supplementary Material, further inquiries can be directed to the corresponding authors.
